# Prediction of Outcome in Patients With Acute Ischemic Stroke Based on Initial Severity and Improvement in the First 24 h

**DOI:** 10.3389/fneur.2018.00308

**Published:** 2018-05-07

**Authors:** Anke Wouters, Céline Nysten, Vincent Thijs, Robin Lemmens

**Affiliations:** ^1^Department of Neurosciences, Experimental Neurology, KU Leuven – University of Leuven, Leuven, Belgium; ^2^Laboratory of Neurobiology, Center for Brain and Disease Research, VIB, Leuven, Belgium; ^3^Department of Neurology, University Hospitals Leuven, Leuven, Belgium; ^4^Stroke Division, Florey Institute of Neuroscience and Mental Health, University of Melbourne, Heidelberg, VIC, Australia; ^5^Department of Neurology, Austin Health, Heidelberg, VIC, Australia

**Keywords:** delta National Institutes of Health Stroke Scale, ischemic stroke, major neurological improvement, modified Rankin scale, National Institutes of Health Stroke Scale, relative reduction National Institutes of Health Stroke Scale, baseline National Institutes of Health Stroke Scale

## Abstract

**Introduction:**

Stroke severity measured by the baseline National Institutes of Health Stroke Scale (NIHSS) is a strong predictor of stroke outcome. Early change of baseline severity may be a better predictor of outcome. Here, we hypothesized that the change in NIHSS in the first 24 h after stroke improved stroke outcome prediction.

**Materials and methods:**

Patients from the Leuven Stroke Genetics Study were included when the baseline NIHSS (B-NIHSS) was determined on admission in the hospital and NIHSS after 24 h could be obtained from patient files. The delta NIHSS, relative reduction NIHSS, and major neurological improvement (NIHSS of 0–1 or ≥8-point improvement at 24 h) were calculated. Good functional outcome (GFO) at 90 days was defined as a modified Rankin Scale of 0–2. Independent predictors of outcome were identified by multivariate logistic regression. We performed a secondary analysis after excluding patients presenting with a minor stroke (NIHSS 0–5) since the assessment of change in NIHSS might be more reliable in patients presenting with a moderate to severe deficit.

**Results:**

We analyzed the outcome in 369 patients. B-NIHSS was associated with GFO (odds ratio: 0.82; 95% CI 0.77–0.86). In a multivariate model with B-NIHSS and age as predictors, the accuracy [area under the curve (AUC): 0.82] improved by including the delta NIHSS (AUC: 0.86; *p* < 0.01). In 131 patients with moderate to severe stroke, the predictive multivariate model was more accurate when including the RR NIHSS (AUC: 0.83) to the model which included B-NIHSS, age and ischemic heart disease (AUC: 0.77; *p* = 0.03).

**Conclusion:**

B-NIHSS is a predictor of stroke outcome. In this cohort, the prediction of GFO was improved by adding change in stroke severity after 24 h to the model.

## Introduction

Stroke is one of the leading causes of disability and death worldwide (1, 2). Patients who are experiencing deficits as a result of an ischemic stroke are worried about their expected outcome. Identifying predictors of functional outcome may be of assistance to physicians when confronted with these concerns from stroke patients. Stroke severity and evolution of the clinical symptoms during the first days after initial presentation are potential valuable predictors of outcome. Improvement in the estimation of clinical outcomes could result in more specific management of stroke rehabilitation as well as clearer informing of patients and their relatives. Multiple studies have focused on the baseline National Institutes of Health Stroke Scale (B-NIHSS) as a predictor of functional outcome (3–8), but only some data are available on the evolution of the National Institutes of Health Stroke Scale (NIHSS) in the first 24 h after stroke onset (6, 9–11). Different parameters have been described to assess this change in stroke severity: Delta NIHSS (B-NIHSS–24 h NIHSS), relative reduction in NIHSS (RR NIHSS; delta NIHSS/B-NIHSS), and major neurological improvement (MNI; NIHSS of 0–1 or ≥8-point improvement at 24 h).

Other identified, independent predictors of outcome are age, sex, mean arterial pressure, history of diabetes, baseline glucose levels, baseline NIHSS score, CT findings, time to treatment and recanalization, current smoking, atrial fibrillation (AF), and statin intake before stroke (3, 12–14).

The aim of this study was to investigate if the prediction of functional outcome after 3 months could be improved by adding the improvement in the first 24 h into a predictive model.

## Patients and Methods

### Study Population

This is an analysis within the prospective Leuven Stroke Genetics Study (15). The study was approved by the local ethics committee of the university hospitals Leuven. All subjects gave written informed consent in accordance with the Declaration of Helsinki. In this retrospective analysis, we included ischemic stroke patients between May 2005 and August 2009, with stroke defined as 24 h lasting symptoms or less than 24 h with a lesion on diffusion weighted imaging, who were admitted to the stroke unit of the University Hospitals Leuven. Mechanical thrombectomy was not performed on patients in this study. Patients were analyzed when NIHSS at baseline, NIHSS during follow-up after 24 h (range 16–50 h), and modified Rankin Scale (mRS) at 3 months could be obtained from the patient files. The following baseline variables were collected: treatment with thrombolysis, age, baseline glycemia, diabetes mellitus, ischemic heart disease, hypertension, AF, hyperlipidemia, active smoking status, and stroke etiology according to TOAST criteria (cardio embolic, large artery atherosclerosis, small artery occlusion, other causes, and undetermined etiology) (16). In a secondary analysis, we pre-specified to analyze this cohort after excluding patients with mild stroke defined as NIHSS at baseline of 0–5 since change in NIHSS at follow-up can potentially more reliably be assessed in patients presenting with a moderate to severe deficit at presentation.

### Parameters of Improvement and Assessment of Functional Outcome at 3 Months

For early neurological evaluation, we sampled the B-NIHSS in the medical records and after 24 h. When the NIHSS after 24 h could not be obtained from the medical files, this was estimated based on the clinical examination at 24 h (17). Delta NIHSS, RR NIHSS, and MNI were calculated. Functional outcome at 3 months was assessed according to the mRS and good functional outcome (GFO) was defined as an mRS of 0–2 (18).

### Statistical Analysis

B-NIHSS and all other collected variables (etiology according to TOAST criteria, treatment with thrombolysis, age, baseline glycemia, diabetes, ischemic heart disease, hypertension, AF, hyperlipidemia, and active smoking status) were analyzed in univariate analysis in a logistic regression with GFO as the dependent variable. Variables associated with outcome in the univariate analysis with a *p*-value of <0.1 were included in a multivariate model. In this multivariate logistic regression, variables were retained if associated with functional outcome with a *p*-value of 0.05 to identify the independent predictors of GFO. Individual associations between delta NIHSS, RR NIHSS, and MNI with GFO were studied and these parameters were separately included in the multivariate model if a *p*-value of <0.1 was obtained in the univariate analysis. Odds ratios (ORs) and 95% confidence intervals (95% CIs) were calculated for these parameters. Receiver operating characteristics (ROC) curve analysis was used to compare the accuracy of the B-NIHSS, delta NIHSS, RR NIHSS, and MNI as predictors of GFO (19). Areas under the curve (AUCs) were calculated and compared using the method presented by Delong et al. (19). If this analysis was significant, the improvement was further quantified by the integrated discrimination improvement (IDI) and the continuous net reclassification improvement (NRI) (20). In addition, the accuracy of the predictive model was determined in the subgroup of patients who received IV thrombolysis. Youden index was calculated to choose the most optimal threshold. Data were analyzed using R statistical software.

## Results

### Baseline Characteristics

We diagnosed ischemic stroke in 491 patients who had been included in the Leuven Stroke Genetics Study. Change in both NIHSS and mRS at 90 days was lacking in four patients. NIHSS at 24 h was not obtained in 111, and an mRS at 90 days was missing in 7. Therefore, we included 369 patients in the final analysis (see Table S1 in Supplementary Material). In a secondary analysis, we excluded mild stroke (baseline NIHSS 0–5) from the cohort, resulting in 131 (36%) patients for this analysis. Table 1 describes the baseline clinical characteristics of the overall population and the cohort of moderate to severe stroke patients.

**Table 1:** Baseline characteristics and outcome of in the overall population and subgroup.

	All stroke (*n* = 369)	Mild to severe stroke (*n* = 131)
Age (median, IQR)	70.2 (57.6–78.1)	72.8 (61.5–79.0)
Male	215 (58.3)	71 (54.2)
Glycemia (mg/dl, median, IQR)	100 (89–115)	104 (92–120)
Stroke etiology		
Cardioembolic	120 (32.5)	48 (36.6)
Small vessel disease	48 (13.0)	10 (7.6)
Large vessel disease	59 (16.0)	28 (21.4)
Other causes	14 (3.8)	3 (2.3)
Undetermined	128 (34.7)	42 (32.1)
Thrombolysis	37 (10.0)	29 (22.1)
Hypertension	232 (62.9)	85 (64.9)
Diabetes	72 (19.5)	30 (22.9)
Smoking	303 (82.1)	93 (71.0)
Atrial fibrillation	85 (23.0)	38 (29.0)
Hyperlipidemia	209 (56.6)	77 (58.8)
Ischemic heart disease	52 (14.1)	24 (18.3)
B-NIHSS (median, IQR)	4 (2–7)	9 (7–15)
Delta NIHSS (median, IQR)	1 (0–3)	4 (1–6)
RR NIHSS (median, IQR)	0.2 (0–0.6)	0.4 (0.1–0.7)
MNI	147 (39.8)	36 (27.5)
GFO	279 (76)	68 (52)

*Data are “*n* (%)” unless defined differently*.

*IQR, interquartile range; B-NIHSS, baseline National Institutes of Health Stroke Scale; NIHSS, National Institutes of Health Stroke Scale; RR NIHSS, relative reduction NIHSS; MNI, major neurological improvement; GFO, good functional outcome*.

### Primary Analysis for the Overall Stroke Cohort

Good functional outcome was present in 279 patients (76%). B-NIHSS was associated with GFO in univariate analysis (OR 0.82; 95% CI 0.77–0.86) as was the change in NIHSS after 24 h when assessed by delta NIHSS and MNI (Table 2). B-NIHSS was a good predictor of GFO with an AUC of 0.78 (95% CI 0.72–0.84). A Youden index of <7 resulted in a sensitivity of 0.81 (95% CI 0.76–0.85) and a specificity of 0.67 (95% CI 0.67–0.76) to predict GFO.

**Table 2:** Logistic regression analysis for GFO in overall stroke cohort (*n* = 369).

	Unadjusted OR (95% CI)	AUC	Adjusted model OR (95% CI)	AUC
B-NIHSS	0.82 (0.77–0.86)	0.78	0.81 (0.76–0.96)^a^	0.82
RR NIHSS	1.23 (0.86–1.76)	0.54	NA	NA
Delta NIHSS	0.94 (0.88–0.99)	0.56	1.34 (1.21–1.49)^b^	0.86
MNI	3.16 (1.88–5.49)	0.63	3.15 (1.65–6.30)^b^	0.84

*B-NIHSS, baseline National Institutes of Health Stroke Scale; RR NIHSS, relative reduction NIHSS; NIHSS, National Institutes of Health Stroke Scale; MNI, major neurological improvement; OR, odds ratio; 95% CI, 95% confidence interval; AUC, area under the curve; GFO, good functional outcome; NA, not applicable*.

*^a^Adjusted for age*.

*^b^Adjusted for B-NIHSS, age*.

**Figure 1:**
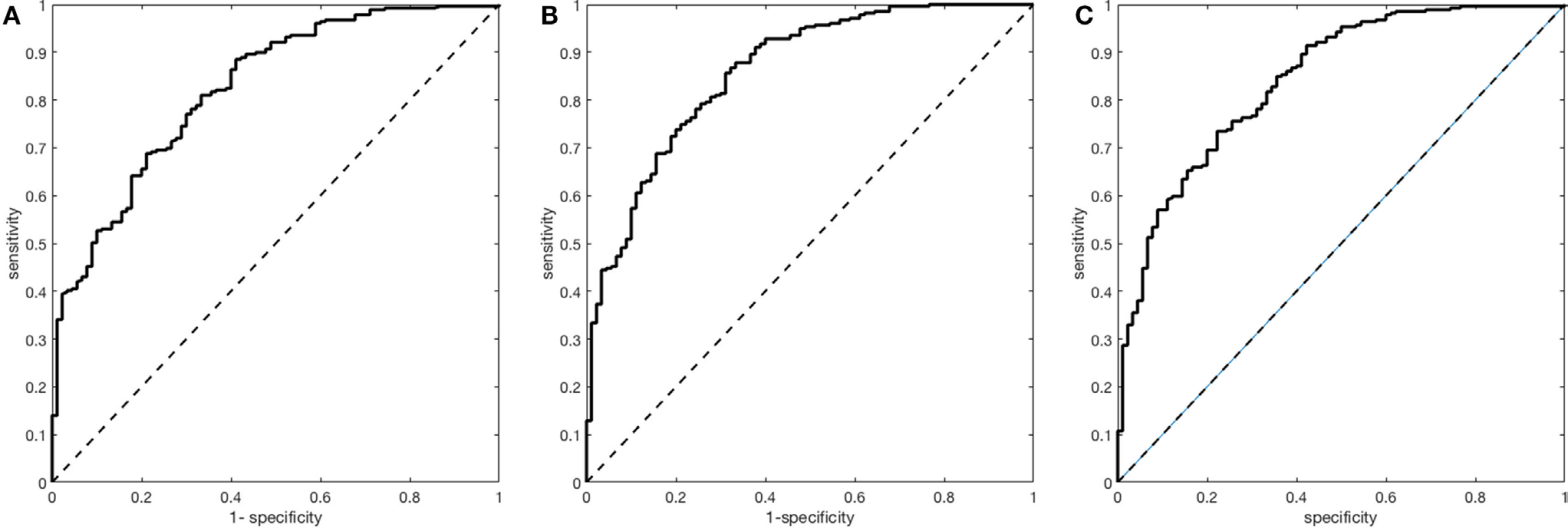
Receiver operating characteristics analyses for the prediction of good functional outcome in the overall stroke cohort. (**A**) Multivariate model with baseline National Institutes of Health Stroke Scale (B-NIHSS) and age [area under the curve (AUC) = 0.82; 95% CI 0.78–0.87]. (**B**) Multivariate model with B-NIHSS, age, and Delta National Institutes of Health Stroke Scale (AUC = 0.86; 0.82–0.90). (**C**) Multivariate model with B-NIHSS, age, and major neurological improvement (AUC = 0.84; 0.80–0.89).

A multivariate prediction model including B-NIHSS and age as predictive variables revealed an AUC of 0.82 (95% CI 0.78–0.87). The accuracy of the model improved by including the delta NIHSS (AUC 0.86; 95% CI 0.82–0.90; NRI 0.78; 95% CI 0.56–1.00; IDI 0.09; 95% CI 0.05–0.12; *p* for difference <0.01) and trended to increase by adding MNI (AUC: 0.84; 95% CI 0.80–0.89; *p* for difference 0.06) (Table 2; Figure 1).

In the subgroup of 37 patients who received intravenous thrombolysis, the accuracy of the multivariate model, including the change in NIHSS assessed with the delta NIHSS, was comparable to that of the total cohort of patients with an AUC of 0.87 (95% CI 0.72–1.00) to predict GFO.

### Secondary Analysis for Patients With Moderate to Severe Stroke on Admission

In the subgroup of 131, GFO was present in 68 (52%) patients. In univariate analysis, B-NIHSS was a predictor of GFO (OR 0.86; 95% CI 0.79–0.93) with an optimal threshold of <10 resulting in a sensitivity of 0.71 (95% CI 0.58–0.81) and a specificity of 0.63 (95% CI 0.50–0.75). In univariate analysis, a relationship between the change in NIHSS after 24 h and GFO was only documented for RR NIHSS (Table 3).

**Table 3:** Logistic regression analysis for GFO in patients with mild to severe stroke patients (*n* = 131).

	Unadjusted OR (95% CI)	AUC	Adjusted model OR (95% CI)	AUC
B-NIHSS	0.86 (0.79–0.93)	0.71	0.86 (0.78–0.94)^a^	0.77
RR NIHSS	6.40 (2.25–20.01)	0.67	15.00 (3.93–70.12)^b^	0.83
Delta NIHSS	1.04 (0.97–1.12)	0.57	NA	NA
MNI	1.97 (0.90–4.43)	0.57	NA	NA

*B-NIHSS, baseline National Institutes of Health Stroke Scale; RR NIHSS, relative reduction NIHSS; NIHSS, National Institutes of Health Stroke Scale; MNI, major neurological improvement; OR, odds ratio; 95% CI, 95% confidence interval; AUC, area under the curve; GFO, good functional outcome; NA, not applicable*.

*^a^Adjusted for age and ischemic heart disease*.

*^b^Adjusted for B-NIHSS, age, and ischemic heart disease*.

**Figure 2:**
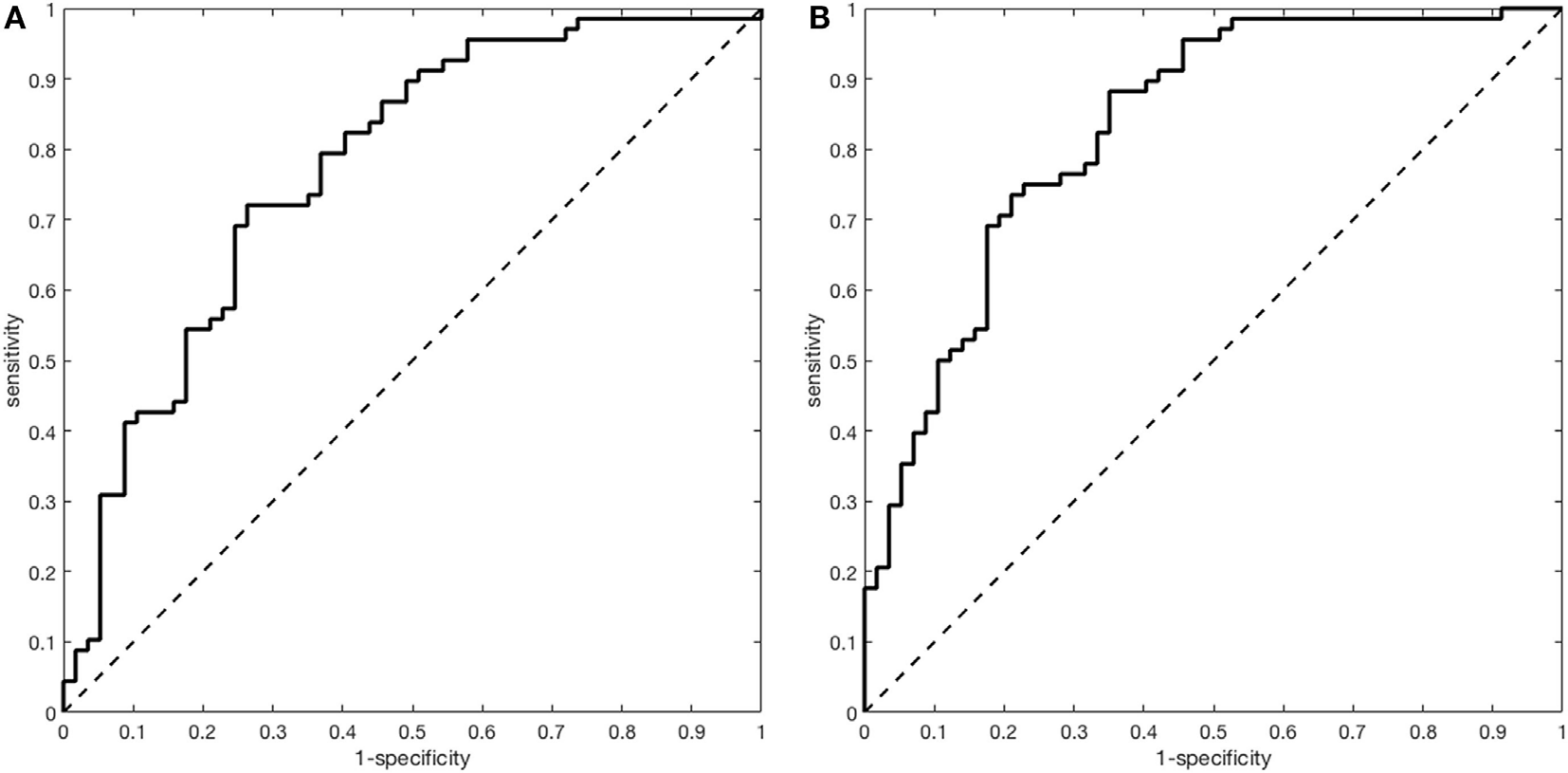
Receiver operating characteristics analyses for the prediction of good functional outcome in the subgroup with mild to severe stroke. (**A**) Multivariate model with National Institutes of Health Stroke Scale (NIHSS), age, and ischemic heart disease [area under the curve (AUC) = 0.77; 95% CI 0.77–0.86]. (**B**) Multivariate model with NIHSS, age, ischemic heart disease, and relative reduction NIHSS (AUC = 0.83; 95% CI 0.76–0.90).

The accuracy of the multivariate model with B-NIHSS, age, and ischemic heart disease to predict GFO (AUC 0.77; 95% CI 0.69–0.86) improved by adding RR NIHSS to the model (AUC 0.83; 95% CI 0.76–0.90; *p* for difference 0.03; NRI 0.52; 95% CI 0.18–0.86; IDI 0.11; 95% CI 0.05–0.16; *p* < 0.01) (Table 3; Figure 2).

## Discussion

Baseline NIHSS is a strong predictor of functional outcome 90 days after stroke. The accuracy of a multivariate predictive model is further improved by including the change in stroke severity over the first 24 h after onset. The change in stroke severity had predictive value in the overall cohort when assessed by the delta NIHSS and in moderate to severe stroke patients the RR NIHSS was identified as a predictor of GFO.

In our study, we replicated the association between B-NIHSS and functional outcome. In the ROC analysis, an NIHSS < 7 was identified as a predictor of GFO which is similar to findings in previous studies (4–7). In our analysis, we dichotomized the B-NIHSS in the predictive model. Categorization of the NIHSS has also been studied in relation to discharge status: with an NIHSS of ≤5 strongly associated with return to home, NIHSS of 6–13 with rehabilitation and NIHSS of >13 with admission in a nursing facility (5).

In addition to baseline stroke severity, the change of symptoms in the first hours after symptom onset has been studied as predictor of functional outcome. In a cohort of patients treated with thrombolytic therapy, the relative reduction of NIHSS 1-day posttreatment was identified as a better prognostic factor compared with the delta NIHSS, most pronounced in patients with greater improvement (10). A study on follow-up of 154 patients revealed an initial NIHSS < 5 and an improvement in neurological symptoms in the first 2 h after stroke to have predictive value for GFO (6). In patients with large vessel occlusion included in the ESCAPE trial and endovascularly treated, improvement in NIHSS within 48 h was a better predictor of outcome than baseline NIHSS (21). The predictive value of change in NIHSS in the early time-interval after stroke might be a reflection of reperfusion (spontaneously or by an intervention), but this could not be analyzed in this cohort since we did not collect data on reperfusion status. In our study of 369 patients, the change in NIHSS showed additional predictive value in a multivariate model by delta NIHSS. In the subgroup of patients who received intravenous thrombolysis, the predictive value of the model was confirmed. Our population was rather skewed to minor strokes, and therefore we performed an additional analysis after excluding minor stroke patients. In this cohort, the change in stroke severity improved the accuracy of the multivariate predictive model when assessed by the RR NIHSS. This suggests that in patients with more severe stroke symptoms the proportion of improvement compared with baseline has more value compared with the absolute change in NIHSS points. This information could be taken into account when informing patients and relatives on prognosis after an acute ischemic stroke.

Age was independently associated with outcome in the overall cohort and after excluding patients with mild stroke. This is a replication of previous findings, although this association could not always be identified after adjusting for other predictive variables (4, 5, 10, 22, 23).

Our study has some limitations. First, this is a retrospective study, and therefore our findings await replication in a prospective cohort. In such a replication study, the differences in predictive value of the various assessments of improvement on the NIHSS are of particular interest. Second, 122 patients were not included in the analysis since data on NIHSS after 24 h and/or mRS at 90 days were lacking. This resulted in a reduced patient number in our analysis and may have resulted in bias. Potentially patients with worse outcomes were not seen after 90 days at the outpatient clinic which may have resulted in the skewing of the cohort toward a less severe phenotype. Third, the B-NIHSS was clearly documented in the medical records, but not always present in the follow-up notes after 24 h. In these patients, we obtained the NIHSS at 24 h based on the neurological examination which was noted. We acknowledge this could have caused a margin of error in the delta NIHSS, the RR NIHSS, and the MNI. Also the delay between symptom onset and arrival at the hospital varied between patients. The B-NIHSS was documented at admission in the hospital, but likely the time-window between symptom onset and hospital arrival differed between patients. Finally, the database was created before the mechanical thrombectomy era. We assume that the same parameters can predict outcome but could not investigate this hypothesis in our patient cohort.

In summary, we aimed to evaluate the prognostic value of initial neurological status and change after 24 h as determined by the NIHSS to predict GFO at 90 days. B-NIHSS was a good predictor of GFO in the overall cohort of stroke patients. The accuracy of a multivariate predictive model improved by adding the change of NIHSS in the first 24 h to the model. The assessment of this change in the NIHSS by absolute or relative measurement might differ based on initial stroke severity.

## Ethics Statement

This is an analysis within the prospective Leuven Stroke Genetics Study (15). The study was approved by the local ethics committee of the university hospitals Leuven. All subjects gave written informed consent in accordance with the Declaration of Helsinki.

## Author Contributions

AW and CN: study concept, design, analysis, and interpretation of data, drafting the manuscript, and revising the manuscript. VT and RL: study concept, design, analysis and interpretation of data, drafting the manuscript, revising the manuscript, and acquisition of data.

## Conflict of Interest Statement

AW: receiving grant from European Union (FP7/2007-2013 nr. 278276 WAKE-UP). CN and VT: none. RL: senior clinical investigator of FWO Flanders.
