# Supplementary material for: Prediction of Outcome in Patients With Acute Ischemic Stroke Based on Initial Severity and Improvement in the First 24 h
Source: Front Neurol. 2018 May 7;9:308. doi: 10.3389/fneur.2018.00308 (PMC5950843; doi:10.3389/fneur.2018.00308)
Supplement: Supplementary file 1 [file table_1.docx]

|  | Excluded (N=122) | Included (N=369) | p-value |
| --- | --- | --- | --- |
| Age (y) | 71.3 (58.6-80.7) | 70.2 (57.6-78.1) | 0.34 |
| Gender (m) | 72 (59.0) | 215 (58.3) | 0.88 |
| B-NIHSS | 5 (2-12) | 4 (2-7) | <0.01 |
| Stroke etiology |  |  |  |
| Cardioembolic | 49 (40.2) | 120 (32.5) | 0.12 |
| Large vessel disease | 17 (16.2) | 59 (16.0) | 0.59 |
| Small vessel disease | 11 (9.0) | 48 (13.0) | 0.24 |
| Other cause | 6 (4.9) | 14 (3.8) | 0.59 |
| Undetermined | 39 (32.0) | 128 (34.7) | 0.58 |
| Treatment with thrombolysis | 17 (16.2) | 37 (10.0) | 0.23 |
| Arterial Hypertension | 75 (61.5) | 232 (62.9) | 0.78 |
| Atrial fibrillation | 39 (32.0) | 85 (23.0) | 0.05 |
| Diabetes | 19 (15.6) | 72 (19.5) | 0.33 |

**Supplementary table 1**

Overview of patient characteristics included vs. excluded in the analysis.

Data are median (IQR) or N (%).

P-values are derived from Mann-Whitney U test (continuous variables) or Chi-Square test (categorical variables).
